# Commencement Exercises

**Published:** 1898-07

**Authors:** 


### COMMENCEMENT EXERCISES.

---

### McGILL UNIVERSITY, VETERINARY DEPARTMENT.

McGILL UNIVERSITY on the afternoon of March 25th increased its long list of graduates by twelve, and at the same time augmented by that number the ranks of those who are entitled to practise the profession of veterinary science.

The function at which this took place was in all respects similar to the many annual convocations of the Faculty of Comparative Medicine and Veterinary Science that have previously taken place in the William Molson Hall.

The exercises were presided over by Mr. George Hague, who was accompanied on the platform by Principal Peterson, Rev. Dr. Clark Murray, Prof. M. C. Baker, Prof. Charles McEachran, Prof. Moyse, Prof. Cox, Prof. Girdwood, Prof. Adami, Prof. Adams, Dr. Morrow, Dr. Bunn, Mr. C. J. Fleet, Mr. W. Vaughan, bursar, and others.

The proceedings were opened with the offering up of prayer by Rev. Dr. Clark Murray, after which Dean McEachran read the faculty lists.

Prizes were then presented to the winners thereof by the chairman, amid applause, viz: Veterinary Medicine and Surgery, W. B. Wallis; Cattle Pathology, W. B. Wallis; Pathology, W. B. Wallis; Materia Medica, W. B. Wallis and W. L. Bell, equal; Anatomy, James McGregor; Physiology, James McGregor; Chemistry, James McGregor; Botany, B. F. Humphries. The dean's silver medal for best general examination in all subjects, W. B. Wallis. Best essay read before the Veterinary Medical Association, J. W. Symes, 1; W. L. Bell, 2; W. B. Wallis, 3. Best essay read before the Society for the Study of Comparative Psychology, J. B. Hart, 1; L. A. Paquin, 2; Junior Class, E. W. Hammond. Examination of Horses for Soundness, W. L. Bell.

Dean McEachran spoke of his late visit to the veterinary colleges, medical colleges, and other scientific institutions of Great Britain, France, Germany, and Denmark. In the course of his remarks he assured his hearers of the unflagging interest that Lord and Lady Strathooma and Mount Royal evinced in McGill University. He referred to the greatly increased interest taken in Canada and Canadian affairs in the Old World, which he attributed largely to the advertisement that the Dominion received through the representation made at Her Majesty's jubilee celebrations. He alluded to the high esteem in which Sir Wilfrid Laurier was held by all classes on the other side of the Atlantic, and spoke of the favorable impression which the members of the British Association, and especially the members of the British Medical Association, had carried away with them after their visit to Canada last year. The Dominion, he said, never had better emigration agents. In conclusion, he urged for consideration the suggestions deducted from the experiments of these older countries, and expressed the hope that before long, instead of the public of Montreal and other large Canadian cities using milk from cows fed upon very indifferent food, confined in filthy, undrained, and unventilated byres, milked in the early hours of the morning, either in the dark or by dim candle-light, without any washing of the udders, without even washing of the milkmen's hands, public opinion would compel the vendors of this important article of human food to handle it in a common-sense and cleanly manner. He also expressed the hope that the consideration of these matters might lead the benefactors of McGill to add to its teaching staff a faculty of agriculture.

The degree of D.V.S. was then conferred on the graduating class, the oath being administered by Mr. Vaughan, and the capping performed by Principal Peterson. Those who received the degree were: W. B. Wallis, J. P. Spanton, W. L. Bell, D. Cullen, L. A. Paquin, B. K. Baldwin, J. B. Hollingsworth, A. W. Cleaves, J. B. Hart, G. H. Lambert, J. G. Pfersick, and G. H. Burke.

The valedictory on behalf of the graduating class was delivered by Dr. W. L. Bell, who spoke of the need of new buildings for the faculty, tendered some advice to his fellow-graduates, urging them not to fall behind in their knowledge, but to take every means in their power to keep up to date in their profession, and concluded by referring in kindly terms to the several members of the teaching staff.

Prof. M. C. Baker, on behalf of the faculty, extended sincere congratulations to the members of the graduating class on the successful termination of the session's work, and expressed the hope that an increased measure of success might attend them in the practical application of the knowledge they had acquired at college. In the treatment of their patients he counselled the graduates ever to bear in mind that although animals lacked the faculty of speech, they were endowed with sensibilities as acute as those who possessed that faculty. He urged them never to inflict unnecessary pain, never to countenance cruelty in others, and never to neglect the use of anæsthetics, local and general, for they were as indispensable in veterinary as they were in human surgery.

Principal Peterson associated himself with the expressions of appreciation that had fallen from the lips of Mr. Hague in reference to the remarks of Dean McEachran, and especially in the direction he had suggested regarding agriculture and farming operations. He expressed the hope that any future extensions of the work of the faculty would always rest on the basis of the present dean's sound scholastic training and on a comprehensive study of the science that underlaid the profession of the veterinarian —biology, comparative pathology, physiology, etc. Increased attention to such requirements would do more than anything else to raise the standard of the veterinary profession and to attract a larger proportion of well-educated young men to its ranks. Addressing all the graduates, he asked them ever to maintain the dignity and to exalt the credit of their alma mater, and never in any way, by word or deed, to tarnish the lustre of the degree which had been conferred upon them that day.

The proceedings closed with the pronouncing of the benediction by Rev. Dr. Clark Murray.

### ONTARIO VETERINARY COLLEGE.

THE students of the Ontario Veterinary College gathered in the lecture hall of the college on the afternoon of March 25th, the occasion being the annual closing exercises and presentation of prizes. Great interest was taken in the proceedings by the students, and as the names of the successful prize-winners were read out they were heartily applauded. On the platform were Hon. E. J. Davis, Provincial Secretary; Dr. J. T. Duncan, Professor A. Smith, F.R.C.V.S.; Thomas Crawford, M. P. P.; Dr. Thorburn, Major Lloyd, V.S., and the Examining Board, J. D. O'Neill, London; W. Shaw, Dayton, Ohio, and Prof. Sisson, President of the Ontario Veterinary Association; C. Elliott, St. Catharine's; Dr. Amyot, of Toronto University.

A short address to the students by Dr. Smith opened the meeting. The Principal said he did not intend to detain them long, as he knew that they were anxious to get home. He congratulated the successful students and urged perseverance on the unsuccessful.

The gold medalist for the year was Mr. C. W. Fisher, of Cabot, Vermont.

After the presentation of prizes had been completed, Professor Smith introduced the Hon. E. J. Davis to the meeting. The Provincial Secretary met with a hearty reception from the students. He expressed his pleasure at what he had seen and at the complete equipment of the college. In the thirty-five years the college has been in existence thousands of scholars had passed their examinations and gone forth to carry on their calling through all parts of the world. In order to become successful, however, the examination was only the beginning not the end of the student's progress. He was sure that Dr. Smith felt pleasure in seeing the many students who had passed through the college and were now eminent in their profession. He advised the students to go ever forward, and he felt sure the students would by their future work realize the importance of their position. Their profession was an important and honorable one, and great responsibilities rested upon them, as they held in their hands millions of dollars worth of property of other people. Some of them might say that their profession was not as good as it used to be, but all businesses had felt hard times. In the veterinary profession, however, the cloud was rolling away. Horses were to-day more valuable than they ever were, and would be still more so. He thought the profession would participate in the good times we were entering upon. With reference to the students from the United States, the speaker was glad to welcome them. He desired the most friendly feeling between Canada, the United States and England. (Applause.) He thought it was a valuable testimony to the success of the college that so many students from the United States selected it. He thought no better compliment could be paid to Dr. Smith and associates. Hon. Mr. Davis closed by wishing all the students prosperity, and wishing Dr. Smith increasing success in the conduct of this important school.

Major Lloyd, one of the examiners, also wished the students every success in their profession. He hoped those from a distance would remember the kindly associations they had met with at the college. He felt that the students this year had done excellent work.

Dr. Thorburn gave a few words of advice to the students, and urged them to uphold the honor of their profession. Do not work too hard, remarked the doctor jocularly, but think of what you are doing, and then they would find that there was always room for one on top. He was very pleased with the result of the examinations, as it showed there was a class of earnest men.

Mr. Charles Elliott, of St. Catharine's, impressed on the minds of the students the importance of looking after the associates they kept, particularly as they were just starting out in life. They should also continue to study and subscribe for such papers as would keep them posted.

Mr. D. L. Devereau, president of the graduating class, then, amid the cheers of the students, presented Professor Smith with a group photograph of the class. He also extended a vote of thanks from the class for the considerate manner with which the students have been treated by Professor Smith and those associated with him. The class also desired to include the examining board in the vote of thanks. At the conclusion the students gave three ringing cheers for Professor Smith.

The latter, in the name of his colleagues and himself, returned thanks for the manner in which Mr. Devereau had referred to those connected with the college, and also for the presentation of the class picture. He hoped to be spared many years to see that picture and many others that graced the walls of the college. In parting with the college he hoped the students would always have a friendly feeling toward the institution. During the time he had been connected with the college very many scholars had come from the other side, and he noticed there was very little difference in matters of education between the two peoples. He believed also that the feeling between Canada and the United States was most friendly. The proceedings closed with three cheers for Hon. Mr. Davis. The latter thanked the students for their kindness, and once more wished them success.

Following is a full list of the graduates:

Walter J. Ackerman, St. Albans, Vermont; William L. Adams, Cabot, Vermont; Daniel Allen, Chesley, Ont.; Arthur E. Atwood, Somerville, Mass.; John D. Bell, Port Elgin, Ont.; Wm. D. Brand, Forrest, Ont.; Charles E. S. Brind, Stourbridge, England; Lawrence Bailey, Rosemont, Ont.; Samuel Caldbick, Brussels, Ont.; George H. Cranston, Attwood, Ont.; William R. Clark, Pettisville, Ohio; E. T. Cunningham, Colbeck, Ont.; Robert B. Coutts, Seattle, Wash.; Harlo R. Clark, Brookfield, N. Y.; J. G. Cruikshank, Deloraine, Man.; William Henry Corey, St. Albans, Vermont; Elwes E. Cary, Orlando, Florida; J. L. Devereau, Waterbury, Conn.; Lawrence T. Dunn, Providence, R. I.; George H. Davidson, Grand Forks, North Dakota; James Dixon, West Liberty, W. Va.; Orvill A. Delong, Florence, Ont.; James Elmer Ellis, Rockport, Ill.; George T. Elliott, Delhi, N. Y.; William E. Fairbanks, Lewiston, Maine; Carl Wallace Fisher, Cabot, Vermont; Thomas I. Fletcher, Ashland, Neb.; P. LeClerc Gauntt, Lumberton, N. J.; Benjamin W. Groff, Massillon, Ohio; George W. Higginson, Hawkesburg, Ont.; John P. Howland, Taunton, Mass.; Walter G. Huyett, Wernersville, Pa.; Fred. M. Hayward, Deansboro, N. Y.; G. Philip Hayter, London, England; George T. Irons, Abilene, Texas; Andrew R. Jordan, Dutton, Ont.; Thorfin Lambrechts, Montevideo, Minn.; Edward Henry Lawley, Brandon, Man.; John S. McIntyre, Sandhill, Ont.; Duncan McKenzie, Teeswater, Ont.; Alexander McGregor, Poland, Maine; Archibald D. McLachlan, Crampton, Ont.; John A. McDonald, Chicago, Ill.; Roderick McDonald, Rosshire, Scotland; G. W. Mackie, Summerside, P. E. I.; Hamlet Moore, Boston, Mass.: Frank J. Neiman, Marshalltown, Iowa; Ion Watson Parks, Burlington, Vermont; John S. Pollard, Ashton, R. I.; Burton W. Powell, Stockdale, Ont.; Horace Panet, Winnipeg, Man.; Louis Pauquette, St. Thomas, Ont.; John Albert Raleigh, Newcastle, Jamaica, W. I.; Thomas Rowland, Toronto, Ont.; Bertsch Royer, Birnamwood, Wis.; J. W. Rutledge, Portage la Prairie, Man.; Edgar Burke Shaw, Summerhill, Ill.; John Short, Grand Valley, Ont.; Harry W. Stedman, Springfield, Mass.; James E. Sexton, Worcester, Mass.; Andreas I. Sorensen, Modesto, Cal.; Edwin R. Stockwell, East Wilson, N. Y.; John Pront Straughan, Jewett Centre, N. Y.; Charles H. A. Stevenson, Carman, Man.; Thomas Sims, Willow City, N. Dak.; James T. Shannon, Lexington, Ky.; Samuel Shepard Treadwell, Brooklyn, N. Y.; Albert G. Van Tine, Mill Grove, N. Y.; Alfred C. Walker, Chichester, England; William M. Wilson, Hartstown, Pa.; John Mason Young, Rowland, Man.

*Prizes Awarded.*

Disease and Treatment: Seniors—C. W. Fisher, 1st prize, silver medal; J. T. Shannon, 2d prize; J. S. Pollard, P. LeClerc Gauntt, A. C. Walker,

3d prize, equal. Honors—A. E. Atwood, W. D. Brand, C. E. S. Brind, L. Bailey, S. Caldbick, H. R. Clark, R. B. Coutts, L. T. Dunn, G. H. Davidson, T. J. Fairbanks, G. P. Hayter, G. T. Irons, T. Lambrechts, E. H. Lawley, Roderick McDonald, D. McKenzie, A. McGregor, A. D. McLachlan, J. A. Macdonald, Hamlet Moore, J. W. Parks, J. A. Raleigh, B. Royer, J. W. Rutledge, E. B. Shaw, E. R. Stockwell, J. P. Straughan, C. H. A. Stevenson, S. S. Treadwell.

Materia Medica: Seniors—L. Bailey, 1st prize; J. T. Shannon, 2d prize; C. W. Fisher, 3d prize. Honors—H. R. Clark, J. L. Devereau, W. G. Huyett, T. Lambrechts, R. McDonald, Hamlet Moore, J. S. Pollard, J. A. Raleigh, J. M. Young.

Chemistry: Seniors—R. McDonald, 1st prize; L. T. Dunn, 2d prize; C. W. Fisher, 3d prize. Honors—L. Bailey, S. Caldbick, James Dixon, J. L. Devereau, G. H. Davidson, E. H. Lawley, Hamlet Moore, D. McKenzie, B. Royer, T. Rowland, C. H. Stevenson, E. R. Stockwell, J. T. Shannon.

Pathology: Seniors—G. T. Irons, J. T. Shannon, 1st prize, equal; C. W. Fisher, 3d prize. Honors—D. Allen, A. E. Atwood, L. Bailey, H. K. Clark, R. B. Coutts, L. Dunn, J. Devereau, James Dixon, G. H. Davidson, Cary E. Elwes, P. L. Gauntt, G. P. Hayter, G. W. Higginson, W. E. Huyett, T. Lambrechts, C. H. Lawley, D. McKenzie, Hamlet Moore, A. D. McLachlan, R. McDonald, J. McIntyre, A. McGregor, C. Owen, J. S. Pollard, J. A. Raleigh, J. W. Rutledge, T. Sims, J. L. Short, H. W. Stedman, A. Sorensen, E. R. Stockwell, S. S. Treadwell, A. C. Walker.

Physiology: Seniors—C. W. Fisher, 1st prize; Roderick McDonald, 2d prize; James T. Shannon, 3d prize. Honors—L. Bailey, G. T. Irons, T. Lambrechts, A. McGregor, D. McKenzie, J. A. Raleigh, J. W. Rutledge, A. C. Walker.

Anatomy: Seniors—C. W. Fisher, 1st prize; G. T. Irons, 2d prize; E. H. Lawley, 3d prize. Honors—D. Allen, W. L. Adams, W. J. Ackerman, L. Bailey, W. D. Brand, S. Caldbick, H. R. Clark, R. B. Coutts, J. L. Devereau, G. H. Davidson, L. T. Dunn, T. Lambrechts, R. MacDonald, D. McKenzie, A. McGregor, A. D. McLachlan, H. Moore, J. S. Pollard, B. Royer, J. W. Rutledge, E. B. Shaw, T. Sims, E. R. Stockwell, S. S. Treadwell, A. C. Walker.

Entozoa: Seniors—Prize, Lawrence Bailey. Honors—A. E. Atwood, W. J. Ackerman, W. D. Brand, E. T. Cunningham, H. R. Clark, A. Cromwell, W. A. Campbell, S. Caldbick, L. T. Dunn, G. H. Davidson, J. Dixon, J. L. Devereau, J. E. Ellis, C. W. Fisher, G. T. Irons, A. Jordan, E. H. Lawley, R. MacDonald, A. D. McLachlan, D. McKenzie, A. McGregor, J. S. McIntyre, H. Moore, J. S. Pollard, T. Rowland, J. A. Raleigh, J. W. Rutledge, J. T. Shannon, E. B. Shaw, J. E. Sexton, E. R. Stockwell, T. Sims, A. J. Sorensen, S. S. Treadwell, A. Walker, A. Young.

Dissected Specimens: Seniors—S. S. Treadwell, gold medal, given by the Toronto Industrial Exhibition Association; R. MacDonald, 2d prize, $20; J. A. Raleigh, 3d prize, $10; W. M. Wilson, 4th prize; J. W. Parks, 5th prize; J. P. Straughan, 6th prize.

Best General Examination: C. W. Fisher, gold medal, given by the Ontario Veterinary Association. Honors—L. Bailey, G. T. Irons, P. L. Gauntt, T. Lambrechts, J. S. Pollard, E. Stockwell, J. T. Shannon.

Primary Examination. Anatomy: Seniors—Wm. A. Campbell, Alex. J.

Cromwell, John Hewins, Robert Lawson, Cranston Owens, Harry P. Reed, Earl B. Truitt.

Materia Medica: Alex. J. Cromwell, John Hewins, Andrew H. Krull, Donald H. McKay, Wm. A. Campbell.

Disease and Treatment: Juniors—W. M. Goff, 1st prize; H. N. MacNeill, 2d prize; J. McDougall, V. J. Andre, 3d prize, equal. Honors—H. W. Carley-Baker, R. Cochrane, J. Corrigan, C. F. Frey, W. J. Guilford, G. Jerome, F. C. Jones, T. B. Judson, T. J. Kirwan, James P. McVicar, A. L. Moore, M. O'Donnell, J. P. Oliveiler, H. J. Pugsley, J. W. Purdy, H. O. Ramsay, A. J. Roll, W. L. Rundle, J. Russell, J. L. Shorey, W. A. Sproule, J. M. Young.

Anatomy: Juniors—J. M. Young, 1st prize; Henry N. MacNeill, W. L. Rundle, 2d prize, equal; J. T. Oliweiler, 3d prize. Honors—V. J. Andre, H. W. Carley-Baker, R Cochran, J. Corrigan, W. M. Goff, G. Jerome, F. C. Jones, L. B. Judson, J. McDougall, A. L. Moore, M. O'Donnell, H. J. Pugsley, J. W. Purdy, Howard Ramsay, A. J. Roll, J. L. Shorey, W. A. Sproule.

Physiology: Juniors—H. N. MacNeill, 1st prize; H. J. Pugsley, 2d prize; George Jerome, 3d prize. Honors—V. J. Andre, John Corrigan, H. W. Carley-Baker, Wesley M. Goff, F. C. Jones, A. Moore, J. F. Oliveiler, J. W. Purdy, W. L. Rundle, H. Ramsay, W. A. Sproule, J. M. Young.

Chemistry: Juniors—A. L. Moore, 1st prize; George Jerome, 2d prize; W. M. Goff, 3d prize. Honors—V. J. Andre, John Corrigan, W. J. Guilford, F. C. Jones, H. J. Pugsley.

Practical Microscopy and Bacteriology: Juniors—Wesley M. Goff, 1st prize; James M. Young, 2d prize; H. J. Pugsley, 3d prize. Honors—V. J. Andre, H. W. Carley-Baker, John Corrigan, Wm. J. Guilford, F. C. Jones, George Jerome, H. N. MacNeill, A. L. Moore, J. F. Oliweiler, J. W. Purdy, W. L. Rundle J. L. Shorey, W. R. Sproule.

### VETERINARY DEPARTMENT, UNIVERSITY OF PENNSYLVANIA, PHILADELPHIA.

On Wednesday, June 8, 1898, in the American Academy of Music, Philadelphia, Pa., the exercises incident to the graduation of the class of 1898 of the Department of Veterinary Medicine, University of Pennsylvania, were held in conjunction with the general commencement exercises of the University.

The graduates of all the departments, led by the University Band, marched from the University to the Academy of Music, arriving there at 10.30 o'clock. The immense building was filled to overflowing with friends of the students and faculties, all eager to witness the ceremonies which should end the student-life of so many hundreds of young men and women.

The highly entertaining programme was begun by the invocation by Rev. Adolph Spaeth, D.D., LL.D. Next was a hymn, "Our Father in Heaven." This was followed by the Commencement Address, which was delivered by Hon. John B. McPherson. Then followed the singing of the University hymn, "Hail! Pennsylvania." Provost Harrison then conferred the degrees upon all the graduates of the various departments of the University. This was an impressive ceremony. Next was the announcement of honors and prizes, followed by presentation of a portrait of the late Dr. Theodore G. Wormley, who for twenty years held the professorship of chemistry and toxicology. The exercises closed by a spirited singing of "The Star-Spangled Banner," and benediction by Rev. Dr. Spaeth.

Music was rendered by the Municipal Band. The degree of Doctor Veterinariæ Medicinæ was conferred upon the following: Stephen Landon Blount, Texas; Guy Edward Chesley, New Hampshire; Albert Edward Cunningham, Nova Scotia; Lewis Debbie Horner, New Jersey; Joseph Johnson, Jr., Pennsylvania; Philip Kerr Jones, Pennsylvania; John Francis McAnulty, Pennsylvania; Stewart White McClure, Pennsylvania; John Henry McNeall, Illinois; Joseph Neilson Megary, Jr., Maryland; John Jacob Repp, Pennsylvania; Ernest Philip Spaeth, Pennsylvania; John Earnest Spindler, New York; Herman Henry Weinberg, Pennsylvania.

Dr. Leonard Pearson, Dean of the Department of Veterinary Medicine, announced the following prizes: The J. B. Lippincott Prize of one hundred dollars to the member of the graduating class who, in the three years spent in the Department of Veterinary Medicine, attains the highest general average in examinations, to John Jacob Repp.

The prize of a book, entitled *Feeds and Feeding*, awarded to the member of the graduating class who passes the best examination in zoötechnics, to John Jacob Repp.

The prize of an écraseur, offered by a friend of the department to the member of the second-year class who passes the best examination in veterinary anatomy, to John Philip Miller.

This is the first year during which Dr. Leonard Pearson has occupied the important position of Dean of the Department of Veterinary Medicine, but even in so short a time rapid strides have been made in advancing the already high standing of the school. Numerous improvements have been made in the buildings and grounds. Through the efforts of Dr. Pearson as State Veterinarian of Pennsylvania and Secretary of the State Livestock Sanitary Board, that Board has established at the Veterinary Department an excellent Laboratory of Bacteriology and Pathology and a building especially built and designed for the purpose of carrying forward experiments relative to various infectious diseases to which domestic animals are subject. These facilities are all at the service of the students of the department, and the veterinarians of Pennsylvania form an adjunct to the State veterinary establishment which is of inestimable value. The course of study has been augmented and revised so as to conform to the latest teachings in the various phases of medical science. A valuable acquisition to the teaching faculty has been made in the appointment of Dr. W. Horace Hoskins as lecturer in veterinary jurisprudence and ethics and business methods.

At eight o'clock in the evening of commencement day the annual banquet of the Alumni Association of the Department of Veterinary Medicine was held in the University restaurant. The committee in charge, composed of Drs. William G. Shaw, C. J. Marshall, and A. F. Schreiber, certainly had the best interests of their brother-alumni in view, as the menu will testify: Salt oysters, sliced tomatoes, queen olives, salted peanuts, consomme de volaille, planked blue-fish au beurre, pomme Julienne, barbecued spring chicken, giblet sauce, waffles, new potatoes, new asparagus, new peas, crab salad, strawberries and ice cream, assorted fancy cakes, fromage de brie, Bent's biscuit, cafe noir. It is needless to say that after such a sumptuous repast all were in a responsive mood when the toastmaster, Dr. John W. Adams, professor of surgery, took charge of the ceremonies. Dr. Adams is a toastmaster of rare ability, and succeeded in calling forth happy responses from the following gentlemen: "The Ideal Veterinarian," Dr. John Marshall; "The Future of Veterinary Science," Dr. Leonard Pearson; "Canine Practice," Dr. Alexander Glass; "Veterinary Ethics," Dr. W. Horace Hoskins, "The Success of a City Veterinarian," Dr. S. J. J. Harger; "Advantages of a Course in Medicine to a Veterinarian," Dr. Conrow; "Country Practice," Dr. W. H. Ridge; "The Veterinarian as a Society Leader," Dr. Robert Formad; "The Use and Abuse of the Bottle," Dr. M. E. Conard; "Reminiscences," Dr. A. F. Schreiber; "Taxidermy and Other Skin Games," Dr. H. D. Martein; "The Veterinarian as a Therapeutist," Dr. E. S. Muir; "The Temptations of a Resident," Dr. W. G. Shaw; "What the Alumni Could Do?" Dr. John J. Repp; "Veterinary Service in the Army," Dr. John P. Turner. Among those present whose names are not given above were Drs. Harry Walter, H. W. Turner, B. F. Senseman, J. M. Mecray, F. H. Mackie, Leroy M. Land, J. Stewart Lacock; also the class of 1898 was present in a body by invitation of the Association, and its members were formally welcomed into membership.

The banquet was a pronounced success in every particular, and everyone felt that another tie had bound him still closer to his alma mater. The alumni, if each of them does his duty, constitute one of the most potent factors in making the success of the school from which they graduated. By making the schools better we raise the standard of the profession. To have a good school it is requisite to have a considerable number of students. No force will avail so much in securing students as the determined effort of the individual alumnus. It is the duty of each one to induce young men who are well qualified to become students in the Department of Veterinary Medicine of the University. Nothing will count for so much as such individual effort.

### NEW YORK STATE VETERINARY COLLEGE.

The commencement exercises of the New York State Veterinary College were held in common with those of other departments of Cornell University at the University Armory, June 16th at 10 A.M., and the Degree of Doctor of Veterinary Medicine conferred by the President of the University upon the following candidates. Henry W. Dustan, Morristown, N. J.; Albert B. Kelly, Albany, N. Y.; Henry Lehrman, Montclair, N. J.; Ray J. Stanclift, Derby, N. Y.

The Horace K. White prize for meritorious work was awarded to R. J. Stanclift.

The graduating class, like that of 1897, was necessarily made up of graduates from other colleges or of students who had taken a part of their course at other institutions. In 1899 the college will begin to graduate students who have taken their complete course in this institution.

The number of students for 1897–1898, as compared with 1896–1897, shows an increase of 55 per cent., while the number of animals presented at the clinic has increased by 100 per cent.

The work accomplished during the year has been highly gratifying alike to faculty and students.

The graduating class of the New York State Veterinary College numbered four of those pursuing their course of study at this institution. The prospects for the school for next year seem to be promising to those engaged in its direction and management. The library of the college has recently been added to by obtaining the entire library of the late John Busted, of New York City, containing many valuable works. The museum has also been added to by a very valuable collection of pathological preparations from the same source. A number of valuable pathological specimens were recently obtained by gift from Dr. W. L. Zuill, of Philadelphia.

### HARVARD UNIVERSITY, SCHOOL OF VETERINARY MEDICINE.

At the commencement exercises of the Veterinary Department, Harvard University, held in connection with the commencement exercises of the other departments of this University, in June, the following gentlemen received the degree of M.D.V.: Ralph Wilson Balkam, Hyde Park, Mass.; Chester Lawrence Blakely, Medford, Mass.; Joseph Horace Dennen, Somerville, Mass.; Leonard Joseph Loewe, Boston, Mass.; Daniel Stephen Joseph Murphy, Cork, Ireland.; Lawrence Locke Peirce, Arlington Heights, Mass.; John William Robinson, Natick, Mass.; William Henry Simpson, Dorchester, Mass.; Windsor Reed Smith, W. Brookfield, Mass.; and Harry Albert Tuttle, Hyde Park, Mass.

